# High-resolution crystal structure of Z-DNA in complex with Cr^3+^ cations

**DOI:** 10.1007/s00775-015-1247-5

**Published:** 2015-02-17

**Authors:** Pawel Drozdzal, Miroslaw Gilski, Ryszard Kierzek, Lechoslaw Lomozik, Mariusz Jaskolski

**Affiliations:** 1 Faculty of Chemistry, A. Mickiewicz University, Umultowska 89b, 61-614 Poznan, Poland; 2 Institute of Bioorganic Chemistry, Polish Academy of Sciences, Noskowskiego 12/14, 61-704 Poznan, Poland

**Keywords:** Z-DNA, Chromium, X-ray crystallography

## Abstract

This work is part of our project aimed at characterizing metal-binding properties of left-handed Z-DNA helices. The three Cr^3+^ cations found in the asymmetric unit of the d(CGCGCG)_2_–Cr^3+^ crystal structure do not form direct coordination bonds with atoms of the Z-DNA molecule. Instead, the hydrated Cr^3+^ ions are engaged in outer-sphere interactions with phosphate groups and O6 and N7 guanine atoms of the DNA. The Cr^3+^(1) and Cr^3+^(2) ions have disordered coordination spheres occupied by six water molecules each. These partial-occupancy chromium cations are 2.354(15) Å apart and are bridged by three water molecules from their hydration spheres. The Cr^3+^(3) cation has distorted square pyramidal geometry. In addition to the high degree of disorder of the DNA backbone, alternate conformations are also observed for the deoxyribose and base moieties of the G2 nucleotide. Our work illuminates the question of conformational flexibility of Z-DNA and its interaction mode with transition-metal cations.

**Electronic supplementary material:**

The online version of this article (doi:10.1007/s00775-015-1247-5) contains supplementary material, which is available to authorized users.

## Introduction

Trivalent chromium, a d^3^ cation, is poorly taken up by living cells. The Cr^3+^ ions are the final product of in vivo Cr^6+^ metabolism. However, Cr^3+^ in contrast to Cr^6+^ can form coordination complexes with macromolecules in the cells [1–3]. In vitro biochemical experiments have shown that exposure of cells to Cr^6+^ yields binary (DNA–Cr^3+^) and ternary (DNA–Cr^3+^–ligand) adducts, DNA crosslinks, as well as oxidative DNA lesions [2, 3]. The DNA–Cr^3+^ complexes are most abundant during in vitro Cr^6+^ reduction and are less mutagenic than the ternary adducts. It was also found that the Cr^3+^ ions decrease the fidelity of DNA polymerase [4]. Several attempts have been made to explain the nature of the DNA–Cr^3+^ interactions. Phosphate groups and/or the N7 guanine atoms have been indicated as the interaction sites in DNA–Cr^3+^ adducts [2, 5–8]. Some nucleotide–Cr^3+^ and DNA–[Cr(Schiff base)] complexes were characterized by different techniques, such as ^31^P NMR, EPR, UV–Vis and IR spectroscopy; however, those studies provided little, if any, precise structural data [9, 10]. Structures of phosphate-based DNA–Cr^3+^ adducts have also been proposed [11]. Despite the interest in DNA–Cr^3+^ interactions in biological systems, the existing literature provides detailed crystallographic structural data for only two, low-resolution DNA–Cr^3+^:DNA polymerase-β complexes, PDB 1zqe (3.7 Å) [12] and 1huz (2.6 Å) [13]. For these two structures, direct interactions of Cr^3+^ with the DNA phosphates were described. In particular, no structures of Z-DNA–Cr^3+^ complexes have been deposited in the PDB [14] or the NDB [15] databases.

Left-handed duplexes of Z-DNA with the self-complementary d(CGCGCG) sequence are remarkable for their potential to form crystals that diffract X-rays to very high resolution. We have used those crystals to obtain accurate information about the geometrical parameters characterizing the coordination of Cr^3+^ ions by left-handed Z-DNA. In the present study, using high-resolution crystallographic data, we describe the coordination geometry of three hydrated Cr^3+^ ions and their outer-sphere interactions with a highly disordered Z-DNA chain in a d(CGCGCG)_2_–Cr^3+^ complex. To the best of our knowledge, a double-conformation guanine base ring is also reported for the first time in any Z-DNA structure.

## Materials and method

### Oligonucleotide synthesis, purification and crystallization

A DNA hexamer with the d(CGCGCG) sequence was synthesized on an Applied Biosystems DNA/RNA synthesizer using phosphoramidite chemistry. The methods of deprotection and purification of the oligodeoxynucleotides have been described previously [16]. A 1.5 mM water solution of the DNA oligonucleotide was annealed at 338 K for 12 min to form a DNA duplex. Single crystals of the DNA were grown at 292 K by the hanging-drop vapor diffusion method by mixing 2 μl of nucleic acid solution and 2 μl of precipitating solution containing 10 % (v/v) (±)2-methyl-2,4-pentanediol (MPD), 40 mM sodium cacodylate, pH 6.0, 80 mM KCl, 12 mM NaCl and 12 mM sperminium tetrachloride. The drops were equilibrated against 0.5 ml of 35 % (v/v) MPD. The crystals appeared within 1 week and grew to the dimensions of 0.2 × 0.1 × 0.1 mm. The best crystals were used for metal ion soaking. For metal soaking, the crystals were placed for several days in 2 μl of the reservoir solution mixed with 2 μl of 5 mM [Cr(H_2_O)_6_]Cl_3_.

### Data collection, structure solution and refinement

X-Ray diffraction data for Cr^3+^-soaked crystals were collected to the resolution of 0.97 Å at the BESSY synchrotron beamline 14.2 in Berlin (Table 1). The crystal was vitrified in a stream of cold nitrogen gas at 100 K. The mother liquor served as a cryoprotectant solution. The data were recorded in four passes, using different crystal-to-detector distances and exposure times to ensure reliable measurement of the high-resolution data and of the strong low-resolution reflections, and to avoid oversaturation of the MAR225 detector. The crystal-to-detector distances, the corresponding maximum resolution, oscillation and the numbers of images in the individual passes were (1) 350 mm, 2.97 Å, 1°, 100; (2) 250 mm, 2.20 Å, 1°, 100; (3) 140 mm, 1.39 Å, 1°, 110; (4) 70 mm, 0.97 Å, 1°, 110, respectively. Detailed statistics of the final merged data set are listed in Supplementary Table S1. The diffraction data were indexed, integrated and scaled using the *XDS* package [17]. The X-ray data statistics are summarized in Table 1. The structure was solved by molecular replacement using *PHASER* [18]. The DNA part of the PDB structure 4hig, corresponding to our earlier model of a d(CGCGCG)_2_–Spk–Mn^2+^ complex [16], was used as a molecular probe. At the initial stages of the refinement, the model was refined using *REFMAC*5 [19] from the *CCP*4 program suite [20]. Later, anisotropic refinement with *SHELXL* [21] was performed using the full resolution of the diffraction data. The anomalous signal, clearly visible in the diffraction data (Supplementary Table S1), reflects the imaginary component (*f′′*) of the anomalous scattering of the chromium cation (1.028 electrons) at the wavelength of 0.9184 Å [22]. Therefore, the structure-factor refinement was carried out with Bijvoet pairs unmerged. The Flack parameter is −0.08(9), confirming the obvious fact that the absolute structure has been determined correctly.

**Table 1:** Data collection and refinement statistics

Data collection	d(CGCGCG)_2_–Cr^3+^
Radiation source	14.2 BESSY Berlin
Wavelength (Å)	0.9184
Temperature (K)	100
Space group	*P*2_1_2_1_2_1_
Cell dimensions (Å)	*a* = 18.14, *b* = 30.44, *c* = 42.94
Resolution range (Å)	24.83–0.97 (1.00–0.97)^a^
Number of reflections	14,037^b^/26,535^c^
Completeness (%)	98.4 (93.2)^c^
Redundancy	2.53 (2.14)^c^
<*I*/σ*I*>	24.85 (2.05)^c^
*R*_merge_^d^ (%)	1.6 (43.2)^c^
Wilson B-factor (Å^2^)	4.87
**Refinement**	
Refinement program	*SHELXL*
Resolution (Å)	24.83–0.97
No. of reflections in working set	13,028^b^/24,677^c^
No. of reflections in test set	1009^b^/1856^c^
*R*/*R*_free_^e^ (%)	14.47^c^/18.49^c^
No. of atoms (nucleic acid/metal/solvent)	240/3/67
<*B*_eq_> (Å^2^) (nucleic acid chain A/B/metal/solvent)	17.5/13.4/13.7/23.7
**R.m.s. deviations from ideal**	
Bond lengths (Å)	0.019
Bond angles (°)	1.93

^a^Values in parentheses correspond to the last resolution shell

^b^Bijvoet pairs merged

^c^Bijvoet pairs separate

^d^ $R_{\text{merge}} = \varSigma_{h} \varSigma_{j} \left| {I_{j}^{h} - \left\langle {I^{h} } \right\rangle } \right| /\varSigma_{h} \varSigma_{j} I_{j}^{h}$, where $I_{j}^{h}$ is the intensity of observation *j* of reflection *h*

^e^ $R = \varSigma_{h} \left| {\left| {F_{\text{o}} } \right| - \left| {F_{\text{c}} } \right|} \right|/\varSigma_{h} \left| {F_{\text{o}} } \right|$ for all reflections, where *F*_o_ and *F*_c_ are observed and calculated structure factors. *R*_free_ is calculated analogously for a random subset of reflections excluded from the refinement

After each round of 50 cycles of CGLS (conjugate-gradient least-squares) minimization, the *COOT* [23] program was used for visualization of electron density maps and manual rebuilding of the atomic model. The occupancies of the DNA moieties in alternative conformations were refined with their sum constrained to unity. Water molecules were added to the model based on *mFo*-*DFc* electron density and stereochemical considerations. There are four close pairs of water molecules with combined occupancy of 1.0. The remaining water sites were classified as fully (11) or partially (52) occupied. Ordered water molecules in the coordination sphere of a given Cr^3+^ ion have the same occupancy as the central atom. Hydrogen atoms were added at their expected positions only to DNA atoms and refined isotropically using the “riding” option.

The model was validated using the free *R* test [24] and the *NuCheck* program [25]. ~7 % of all reflections were selected at random and set aside for *R*_free_ calculations. The test reflections were included in the work set during the final rounds of refinement. Most of the stereochemical restrains for the DNA moieties were taken from Clowney et al. [26], Gelbin et al. [27] and Parkinson et al. [28]. However, the target values for the C4–N9–C1′ [129.0(7)°] and C8–N9–C1′ [124.5(7)°] exocyclic angles in *syn* purines, which seem to be inadequate in the above dictionaries, were taken from the ultrahigh-resolution Z-DNA–Spm structure (0.55 Å; PDB 3p4j) reported by Brzezinski et al. [29]. The 3p4j structure was also used for restraining the O–P–O valence angles in a conformation-dependent manner [30]. At the conclusion of the refinement, one cycle of full-matrix least-squares minimization was calculated with all reflections, but no restraints included to estimate the standard uncertainties (e.s.u.) of the structural and all refined parameters.

**Fig. 1:**
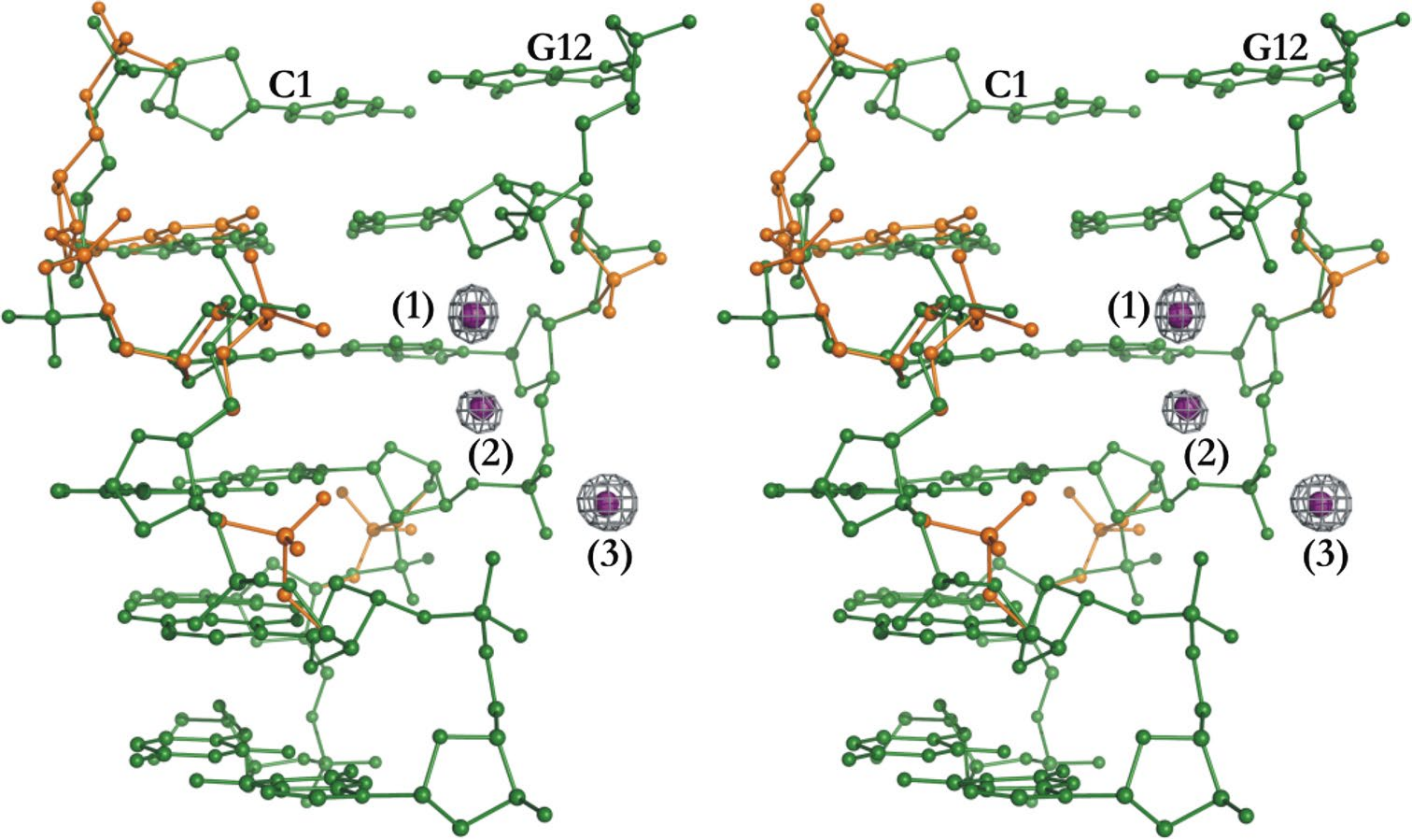
Stereoview of the d(CGCGCG)_2_–Cr^3+^ structure with anomalous difference map for the three Cr^3+^ cations (*purple spheres*). The map is contoured at 6.5σ. Note the alternate conformations (I, *green*; II, *orange*) along the DNA chains

The coordinates of the Cr^3+^(1), Cr^3+^(2) and Cr^3+^(3) ions coincide with the most prominent peaks in the anomalous difference Fourier map at 15.0, 10.0 and 16.1σ, respectively (Fig. 1). The pseudorotation amplitude (*τ*_m_) and phase angle (*P*) of the DNA furanose rings as well as their deviations from the ideal (cyclopentane) model were calculated by the method of Jaskolski [31] (Supplementary Table S2). The Z-DNA helical parameters were calculated using 3*DNA* [32] and the figures were generated with *PyMol* [33].

## Results

### Quality of the results

The estimated standard uncertainties of fully occupied DNA atomic positions are 0.017–0.078 Å (C atoms), 0.015–0.047 Å (N), 0.011–0.129 Å (O) and 0.009–0.018 Å (P). The positional uncertainties for the metal cations are 0.012, 0.021 and 0.012 Å for Cr^3+^(1), Cr^3+^(2) and Cr^3+^(3), respectively.

### Overall structure and helical parameters

The asymmetric unit of the complex contains one d(CGCGCG)_2_ Z-DNA duplex. The d(5′-CGCGCG-3′) nucleotides of chain A are numbered 1–6, and the complementary d(3′-GCGCGC-5′) nucleotides of chain B are numbered 12–7. The phosphate groups are only present at the internucleotide linkages. The free 5′-OH and 3′-OH groups are not phosphorylated. The general structural parameters of the DNA double helix in the complex classify it into the Z-DNA family of duplexes. The major (I) and minor (II) conformations of the G2 nucleotide have C3′*-*endo sugar puckers, with pseudorotation angles of 35.7 and 12.6°, respectively. Different pseudorotation angles are also observed at C3, *P* = 154.7° (I) and *P* = 144.3° (II), although both conformations are in the C2′-endo range. The sugar pucker of C11 has the pseudorotation angle of 142.8°, classifying it as C1′-exo. The 3′-terminal nucleotides G6 and G12 have C2′-endo sugar pucker. A comparison of the helical parameters and geometry of the present Z-DNA–Cr^3+^ complex and the previously reported Z-DNA structures is included in Supplementary Table S3.

### Disorder of the DNA molecule

**Fig. 2:**
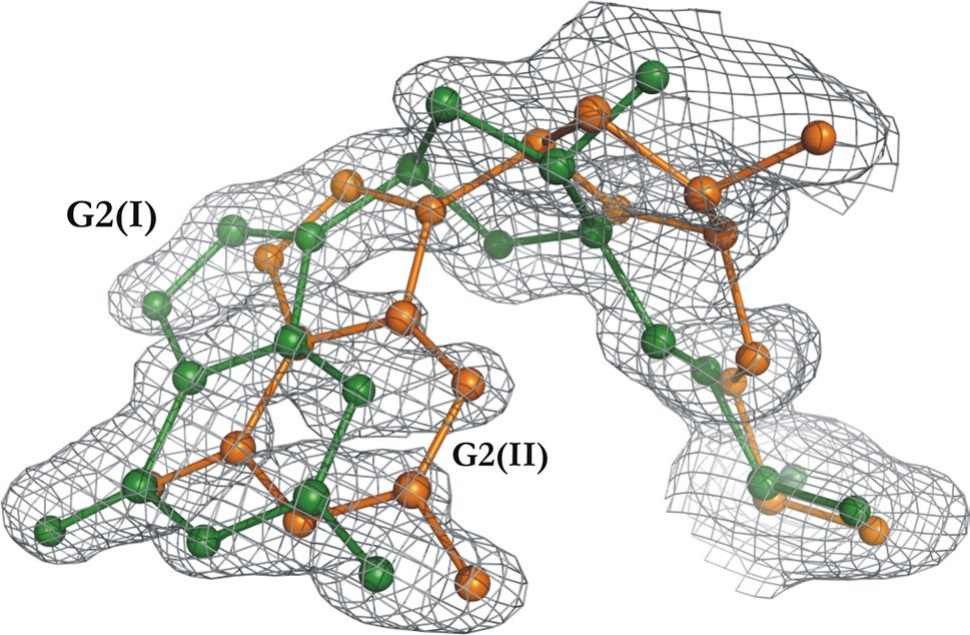
A portion of the Z-DNA duplex in the d(CGCGCG)_2_–Cr^3+^ complex at the G2 nucleotide with two alternative conformations (I, *green*; II, *orange*) shown in 2*mFo*-*DFc* map contoured at 1.0σ

A characteristic feature of the d(CGCGCG)_2_–Cr^3+^ structure is a high degree of disorder of the DNA chains. Double conformations are observed at the internucleotide phosphate linkages C1–G2, G2–C3, C3–G4, G4–C5, G8–C9 and G10–C11 (Fig. 1). These alternate conformations, designated as I/II, have occupancies of 54/46, 54/46, 69/31, 44/56, 66/34 and 78/22 %, respectively. In addition, the electron density map clearly shows alternative positions for both the base and deoxyribose rings of the G2 nucleotide (Fig. 2). The base atoms of G2 are translated by up to 1.66 Å between the two states. The hydrogen bond lengths for the two pairs, G2(I)·C11 and G2(II)·C11, differ from the distances in the canonical Watson–Crick C·G base pair [34]. The N2···O2, N1···N3 and O6···N4 H-bond distances are shorter in the G2(I)·C11 pair [2.742(28), 2.702(28) and 2.517(32) Å] and longer in the G2(II)·C11 pair [3.006(27), 3.25(14) and 3.374(90) Å].

The disordered phosphate groups have Z_I_ and Z_II_ conformation for the alternative positions. Z_II_ conformation of the nucleotide fragments can be attributed to G2(I) (*ζ* = 59.2°), G4(I) (61.9°), G8(II) (73.3°) and G10(I) (74.7°). The Z_II_ conformations are stabilized by hydrogen bonds between a phosphate oxygen atom and water molecules form the coordination spheres of the Cr^3+^ cations, as well as by contacts with symmetry-related Z-DNA helices in the crystal lattice.

The presence of the double-conformation G2 nucleotide causes significant distortions of the helical parameters for the two dinucleotide pairs, C1G2–C11G12 and G2C3–G10C11, as well as of the average values for the entire duplex (tip and inclination; Supplementary Table S2) [32]. The helical parameters shift/tilt/*x*-displacement have the following values: −0.04 and 0.23 Å/0.84 and 2.39°/−48.89 and −31.99 Å, for the pairs of dinucleotides C1G2(I)–C11G12 and C1G2(II)–C11G12, respectively. For the dinucleotide pair C3G4–C9G10, there is a difference in the width of the minor groove (after subtraction of 5.8 Å for the combined radii of the phosphorus atoms) between conformations Z_I_ (7.2 Å) and Z_II_ (6.0 Å).

### Coordination of the Cr^3+^ cations and crystal packing

The Cr^3+^(1), Cr^3+^(2) and Cr^3+^(3) ions have partial occupancies of 35, 26 and 40 %, respectively (Fig. 1). The Cr^3+^(1) and Cr^3+^(2) cations, which are unexpectedly only 2.354(15) Å apart, were treated as separate individual ions during the refinement. It was not possible to unambiguously correlate their partial populations as alternatives within the networks of partially occupied water and disordered DNA fragments. In conclusion, despite their partial occupancies, the possibility of a direct interaction between the Cr^3+^(1) and Cr^3+^(2) ions in the crystal lattice cannot be excluded, especially in view of the bridging water molecules. A similarly short contact [2.394(1) Å] between Cr^3+^ ions was found in a μ-methylene complex, as reported by Noh et al. [35].

**Fig. 3:**
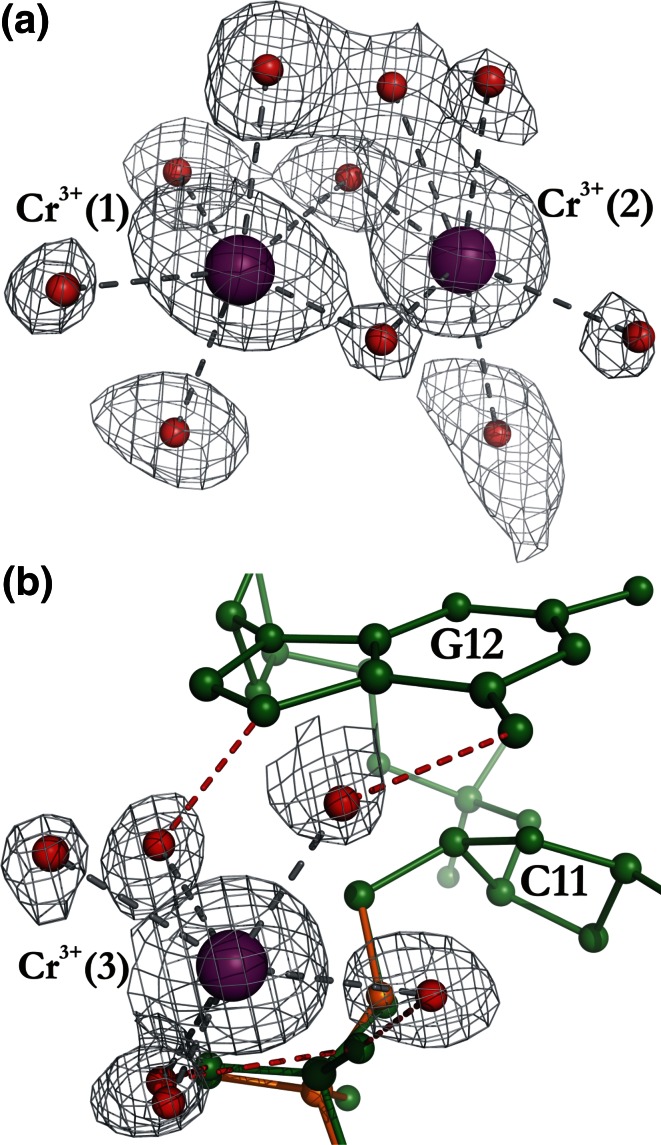
**a** The coordination spheres of the hydrated Cr^3+^(1) and Cr^3+^(2) ions. The 2*mFo*-*DFc* map is contoured at the 1.0σ level. **b** The distorted square pyramidal coordination sphere of the Cr^3+^(3) cation. The 2*mFo*-*DFc* map is contoured at the 1.0σ level. Water molecules are represented by *red spheres*. The coordination and hydrogen bonds are represented by *gray* and *red dashed lines*, respectively

The coordination spheres of Cr^3+^(1) and Cr^3+^(2) contain six water molecules each (Fig. 3a; Table 2). The Cr^3+^(1) and Cr^3+^(2) ions are bridged by three water molecules from their coordination spheres, one of which (Wat1) is split into two sites. The hydration patterns of Cr^3+^(1) and Cr^3+^(2) are irregular and difficult to define. The detailed geometry of the coordination spheres of Cr^3+^(1) and Cr^3+^(2) is summarized in Table 2. The water molecules from the hydration spheres of Cr^3+^(1) and Cr^3+^(2) form numerous hydrogen bonds with the DNA atoms. The water molecules from the coordination sphere of Cr^3+^(1) are hydrogen bonded to OP2_G4(I), OP1_G12, OP2_G12, and to N7_G10^**i**^ (*x* − 1, *y*, *z*), OP2(I)_C11^**ii**^ (*x* − 1/2, 3/2 − *y*, 1 − *z*), OP1(II)_C11^**ii**^ from symmetry-related molecules. The hydration sphere of the Cr^3+^(2) ion is engaged in contact with OP1_G6, and with N7_G10^**i**^, OP1(II)_C9^**i**^, OP1(II)_C11^**ii**^, OP2(I)_C11^**ii**^ from symmetry-related molecules. Similarly to Cr^3+^(1) and Cr^3+^(2), also the Cr^3+^(3) cation is not coordinated directly by atoms of the nucleic acid. The coordination sphere of Cr^3+^(3) is square pyramidal, with five water molecules, one of which (Wat37) is split into two sites (Fig. 3b). The Cr^3+^–OH_2_ bond length ranges for the metal cations 1/2/3 are, respectively, 1.59(4)–2.35(4)/1.73(6)–2.41(3)/1.83(5)–2.65(5) Å (Table 2). Water molecules as ligands of Cr^3+^(3) form hydrogen bonds with OP1_G6, OP2_G6, and with OP1(I)_C11^**ii**^, OP2(I)_C11^**ii**^, O6_G12^**ii**^, N7_G12^**ii**^ from a symmetry-related molecule. The distances from the Cr^3+^(3) cation to Cr^3+^(1) and Cr^3+^(2) are 7.007(11) and 6.017(15) Å, respectively. It should be noted that the hydrated Cr^3+^ cations in the Z-DNA–Cr^3+^ complex interact via their hydration shells with phosphate groups of the DNA fragments in double conformation.

**Table 2:** Coordination geometry (Å, °) around the Cr^3+^ cations

Cr^3+^ (1)	Distance	Angle					
Wat30	1.59 (4)						
Wat1 (II)	1.95 (3)	105 (2)					
Wat24	1.95 (2)	95 (2)	155 (1)				
Wat8	2.00 (2)	141 (2)	65 (1)	90 (1)			
Wat45	2.03 (5)	112 (3)	99 (1)	86 (1)	107 (2)		
Wat31	2.35 (4)	92 (2)	63 (1)	103 (1)	50 (1)	154 (1)	
Cr^3+^ (2)	2.354 (15)	47 (2)	81 (1)	104.3 (8)	94 (1)	156 (1)	44.7 (9)
	Cr^3+^ (1)	Wat30	Wat1 (II)	Wat24	Wat8	Wat45	Wat31

Cr^3+^ (2)	Distance	Angle					
Wat30	1.73 (6)						
Wat31	1.79 (4)	110 (2)					
Wat1 (I)	1.90 (6)	96 (2)	52 (2)				
Wat66	2.03 (7)	89 (2)	88 (2)	36 (2)			
Wat27	2.03 (5)	80 (2)	168 (2)	123 (2)	87 (2)		
Wat40	2.41 (3)	101 (2)	101 (2)	152 (2)	163 (2)	82 (2)	
Cr^3+^ (1)	2.354 (15)	43 (1)	68 (2)	68 (2)	86 (2)	122 (2)	110.6 (8)
	Cr^3+^(2)	Wat30	Wat31	Wat1(I)	Wat66	Wat27	Wat40

Cr^3+^ (3)	Distance	Angle				
Wat37 (II)	1.83 (5)					
Wat64	1.90 (4)	161 (2)				
Wat36	1.95 (3)	89 (2)	84 (2)			
Wat48	2.06 (6)	107 (3)	86 (3)	66 (3)		
Wat37 (I)	2.65 (5)	23 (3)	140 (2)	69 (2)	107 (3)	
Wat38	2.43 (5)	85 (2)	78 (1)	94 (1)	157 (3)	76 (2)
	Cr^3+^(3)	Wat37 (II)	Wat64	Wat36	Wat48	Wat37 (I)

The crystals of the Z-DNA–Cr^3+^ complex belong to space group *P*2_1_2_1_2_1_ and have the less common B type of crystal packing of Z-DNA structures (also called pure-spermine type), as reported for the first time by Egli et al. [d(CGCGCG)_2_–Spm; PDB 1d48] [36]. As described in detail by Brzezinski et al. [29], d(CGCGCG)_2_ Z-DNA crystallizes into two polymorphic forms (A and B) of the same space group and cell parameters, but with different crystal packing. A detailed discussion of the influence of metal and/or polyamine cations, and disorder of Z-DNA on crystal packing can be found in Brzezinski et al. [29], Schuerman et al. [37] and Harper et al. [38].

### Stacking interactions

Except for the G2(II)·C11 base pair, the DNA duplex in the d(CGCGCG)_2_–Cr^3+^ complex has the structure of stacking interactions typical of the Z-DNA form. For the G2(I)·C11 and G2(II)·C11 base pairs, important stacking differences are noted when compared with other duplexes with the d(CGCGCG)_2_ sequence. The overlap areas of the guanine ring G2 (including the exocyclic N and O atoms) in conformations I and II over the C3 base are 3.75 and 5.99 Å^2^, respectively. For the previously described d(CGCGCG)_2_–Spk–Mn^2+^ (PDB 4hig), d(CGCGCG)_2_–Spk–Zn^2+^ (4hif), d(CGCGCG)_2_–Put^2+^–K^+^ [39] and d(CGCGCG)_2_–Spm (3p4j) complexes, the analogous values of the overlap area for the same bases were 4.36, 5.24, 4.76, and 4.76 Å^2^, respectively.

### Hydration

The d(CGCGCG)_2_–Cr^3+^ complex is similar to other high-resolution structures of Z-DNA with regard to the architecture of the hydration shell, which comprises a complicated network of hydrogen bonds that stabilize the molecular packing in the crystal. The asymmetric unit contains 67 water sites. The characteristic features of hydration geometry noted in other Z-DNA crystals, such as the spine of hydration [40], water molecules between N2_G and phosphate O atoms, two water molecules H-bonded to each O6_G group, or absence of water molecules H-bonded to the N3_G atoms, are also found in the present Z-DNA–Cr^3+^ complex. The positions of two disordered water molecules, Wat2 and Wat53, are correlated with the alternate conformations of the DNA chain between the G2-C3 and G8-C9 nucleotides, respectively. The G2(I)·C11 and G2(II)·C11 base pairs have different schemes of hydration. There are no N2···Wat interactions for the G2(I) conformation. Atom O6_G2(I) forms hydrogen bonds with Wat10 [3.182(48) Å] and Wat65 [2.560(72) Å]. Atom N7_G2(I) is H-bonded to Wat10 [3.143(62) Å]. In the alternative conformation, the N2_G2(II) atom forms a hydrogen bond with Wat22 [2.862(45) Å], which bridges it with OP2_C3(II) [2.794(27) Å]. There is also a weak H-bond interaction N2_G2(II)···Wat68 [3.366(49) Å].

## Discussion

The high-resolution crystal structure of the d(CGCGCG)_2_–Cr^3+^ complex presented in this work shows the presence of three Cr^3+^ ion sites per one Z-DNA duplex, which do not form any direct coordination bonds with either the guanine N/O atoms or the phosphate groups of the DNA. Only water-mediated contacts between the nucleic acid and the Cr^3+^ cations are observed. The degree of disorder of the DNA strands in the Z-DNA–Cr^3+^ complex is comparable with that in the recently presented metal- and polyamine-cation free Z-DNA hexamer (PDB 3wbo) [41] and dodecamer (4ocb) [42] structures, and even higher than in the Z-DNA complexes with Mn^2+^ (4hig) and Zn^2+^ (4hif) reported by Drozdzal et al. [16] or in d(CGCGCA):d(TGCGCG)–[Ru(NH_3_)_6_]^3+^ (2hto) [43]. The high degree of disorder in this and similar Z-DNA structures is in stark contradiction to the rigidity of the same chemical molecule (crystallized without metal cations) reported by Brzezinski et al. [29]. In view of the accumulated facts, it seems that the rigidity observed in that ultrahigh-resolution structure (PDB 3p4j) was a fortuitous exception rather than a rule. In addition to alternative positions of the backbone phosphate groups, the present electron density maps also clearly show a double conformation of the G2 nucleoside. The alternate conformations of G2 in the Z-DNA–Cr^3+^ structure resulted in deviations from typical hydrogen bonding and stacking interaction observed for the G2C3–G10C11 base pairs in similar d(CGCGCG)_2_ structures. Analysis of the positions of the base rings of G2(I) and G2(II) indicates a tendency to keep the DNA strands in favorable contacts with water molecules. The alternative positions of the G2 nucleotide do not affect the orientation of the complementary C11 base. The *mFo*-*DFc* map for the C11 nucleotide does not indicate any peaks (even at the 2.5σ level) to suggest possible alternate positions of the cytosine base, which could be correlated with the G2(I)/(II) conformations. This observation is in contrast to findings based on a high-resolution (0.96 Å) B-DNA–Mg^2+^ structure with full base-pair positional heterogeneity (PDB 3u89) [44]. The present structure of the d(CGCGCG)_2_–Cr^3+^ complex confirms the inherent polymorphism in the positions of the DNA atoms and suggests that complementary bases can move independently within the DNA scaffold. Thus, the tendency to maintain the exact Watson–Crick base-pairing geometry with both G2 conformations is not the predominant factor for the C11 nucleotide and many other factors, such as stacking interactions, water and/or ligand interactions, as well as crystal packing, should be taken into account when considering the energy balance of a single nucleotide conformation. Moreover, the stable position of the base ring of C11 within the G2(I)/(II)·C11 base pair may confirm the crucial role played by water molecules in DNA stabilization [45].

The crystal structure of the d(CGCGCG)_2_–Cr^3+^ complex analyzed in this study has the same packing mode of the Z-DNA helices as the d(CACGIUG)_2_–[Co(NH_3_)_6_]^3+^ (PDB 1omk) [37] and d(TGCGCA)_2_–[Co(NH_3_)_6_]^3+^ (362d) structures [38]. Although sperminium tetrachloride was present in the crystallization conditions, the spermine^4+^ tetracation has not been identified in the electron density maps of the d(CGCGCG)_2_–Cr^3+^ crystal. This suggests either a complete disorder of the entire spermine^4+^ molecule, or its total absence in the crystal structure.

## Conclusions

Our studies of the d(CGCGCG)_2_–Cr^3+^ complex indicate that Z-DNA, especially in complexes with metal cations, has a large potential for conformational flexibility. The present structure, as well as other examples deposited in the PDB, strongly supports the notion that Z-DNA helices should not be regarded as having extremely regular and rigid stereochemistry, in contradiction to the suggestion made by Brzezinski et al. [29]. The d(CGCGCG)_2_–Cr^3+^ structure is an excellent illustration of the flexibility of the Z-DNA molecule, visible in the adoption of multiple conformations (by the phosphate groups and the G2 nucleotide), in response to changes in its electrostatic and hydration environment, caused by the introduction of hydrated metal complexes.

## Electronic supplementary material

Below is the link to the electronic supplementary material.

Supplementary material 1 (PDF 120 kb)
Atomic coordinates and structure factors for the d(CGCGCG)_2_–Cr^3+^ complex have been deposited in the Protein Data Bank with accession code 4r15.

## Abbreviations

Spm: Spermine
Spk: Sperminium tetracation
Put^2+^: Putrescinium dication
