# Supplementary material for: High-resolution crystal structure of Z-DNA in complex with Cr3+ cations
Source: J Biol Inorg Chem. 2015 Feb 17;20(3):595–602. doi: 10.1007/s00775-015-1247-5 (PMC4381091; doi:10.1007/s00775-015-1247-5)
Supplement: Supplementary file 1 — Supplementary material 1 (PDF 120 kb) Atomic coordinates and structure factors for the d(CGCGCG)2–Cr3+ complex have been deposited in the Protein Data Bank with accession code 4r15. [file 775_2015_1247_MOESM1_ESM.pdf]

Supplementary Material

High-resolution crystal structure of Z-DNA in complex with Cr^{3+} cations

Pawel Drozdzal[a], Miroslaw Gilski[a,b], Ryszard Kierzek[b],
Lechoslaw Lomozik[a], Mariusz Jaskolski[*a,b]

[a] Faculty of Chemistry, A. Mickiewicz University, Poznan, Poland
[b] Institute of Bioorganic Chemistry, Polish Academy of Sciences, Poznan, Poland

Supplementary Table S1. Data collection and refinement statistics for the d(CGCGCG)$_2$–Cr^{3+} structure (unmerged Bijvoetr pairs).

Resolution (Å)	Number of reflections		Completeness (%)	R_{merge} (%)	$\langle I/\sigma I\rangle$	R-meas(a) (%)	Anomalous CC(b) (%)
	observed	unique					
4.35	1158	291	98.3	1.3	97.98	1.5	73
3.08	2382	547	98.9	1.3	95.82	1.4	58
2.51	3947	707	100.0	1.4	96.92	1.6	64
2.18	3939	855	99.9	1.6	81.97	1.8	69
1.95	3259	941	99.2	1.7	64.04	2.0	53
1.78	2956	1044	98.2	1.7	53.82	2.1	45
1.64	2767	1131	98.3	1.8	45.94	2.3	37
1.54	2818	1222	98.6	1.8	38.90	2.4	40
1.45	2976	1300	98.3	2.4	30.80	3.1	29
1.38	3153	1376	98.7	3.4	24.11	4.4	24
1.31	3271	1435	99.3	4.3	19.97	5.6	13
1.26	3439	1531	99.0	4.6	17.68	5.9	11
1.21	3563	1578	99.8	6.6	12.87	8.6	10
1.16	3701	1667	99.6	7.1	12.05	9.3	7
1.12	3811	1718	99.6	8.9	9.54	11.5	16
1.09	3902	1776	99.2	11.9	7.26	15.4	9
1.06	3935	1804	98.6	16.9	5.17	21.9	3
1.03	4055	1876	98.0	22.1	3.91	28.8	-2
1.00	4013	1869	97.5	33.4	2.64	43.4	2
0.97	3987	1867	93.2	43.2	2.05	56.5	8
**total**	**67032**	**26535**	**98.4**	**1.6**	**24.85**	**1.9**	**17**

(a) Redundancy independent R-factor (intensities) [1].

(b) Correlation coefficient between two random subsets of anomalous intensity differences [1].

Supplementary Table S2. Sugar and phosphate backbone torsion angles for the d(CGCGCG)$_2$–Cr^{3+} structure, with standard uncertainties in parentheses.

d(CGCGCG)$_2$ in the Cr^{3+} complex												
Angle	C1(I)<br>C1(II)	C3(I)<br>C3(II)	C5(I)<br>C5(II)	C7	C9(I)<br>C9(II)	C11(I)<br>C11(II)	G2(I)<br>G2(II)	G4(I)<br>G4(II)	G6	G8(I)<br>G8(II)	G10(I)<br>G10(II)	G12
α	-	158(8)<br>-160(10)	171(6)<br>-158(5)	-	-150(3)<br>163(9)	171(9)<br>-177(8)	62(18)<br>74(18)	71(15)<br>81(35)	84(3)	61(2)	67.7(9)	84(1)
β	-	151(16)<br>-120(22)	149(3)<br>-126(3)	-	-132(2)<br>153(6)	145(1)<br>-136(6)	-174(12)<br>-175(12)	179(10)<br>174(26)	-179(1)	-176(1)	-168.7(7)	-177.6(9)
γ	49(5)	62(31)<br>51(38)	52(3)<br>63(3)	45(2)	61(3)<br>56(6)	51(2)<br>72(4)	-166(10)<br>163(11)	176(11)<br>176(35)	-170(2)	178(1)	-177.4(9)	-173(1)
δ	147(7)<br>143(8)	138(21)<br>136(26)	145(1)	143(2)	151(1)	137.4(9)	93(5)<br>95(6)	95(14)<br>92(30)	152(2)	93(3)<br>89(5)	88(2)<br>96(8)	151(1)
ε	-88(8)<br>-102(6)	-94(21)<br>-93(27)	-97(1)	-94(2)	-98(1)	-99.2(9)	-167(4)<br>-136(3)	-174(6)<br>-104(11)	-	-105(4)<br>173(8)	-174(3)<br>-112(14)	-
ζ	70(11)<br>87(11)	76(14)<br>64(21)	65(2)	79(2)	78(1)	61.6(9)	59(6)<br>-55(4)	62(9)<br>-74(14)	-	-78(4)<br>73(11)	75(3)<br>-55(18)	-
χ	-154(1)	-156.4(7)	-156.8(8)	-153.5(8)	-160.7(7)	-153.2(9)	50(15)<br>66(5)	60(2)	70(1)	55(2)	56(1)	70(1)
ν0	-25(3)	-25(14)<br>-36(18)	-27(1)	-30(2)	-24(1)	-34(1)	-7(9)<br>2(5)	-4(2)	-23(1)	-5(2)	-8(1)	-22(1)
ν1	37(3)	35(12)<br>44(11)	39(2)	37(2)	39(1)	41(1)	-9(8)<br>-13(4)	-11(2)	37(2)	-10(2)	-10(1)	37(1)
ν2	-36(3)	-31(20)<br>-35(20)	-35(1)	-31(2)	-38(1)	-32(1)	19(6)<br>18(5)	20(3)	-35(2)	20(2)	22(1)	-38(1)
ν3	23(3)	18(25)<br>15(29)	20(1)	15(2)	24.2(9)	12(1)	-24(4)<br>-17(6)	-23(2)	22(2)	-23(1)	-28(1)	26(1)
ν4	1(3)	4(24)<br>12(28)	4(1)	10(2)	-0.1(9)	13(1)	20(6)<br>10(6)	17(2)	0(2)	18(1)	22(1)	-3(1)
Pseudorotation parameters:												
P	160.6(8)	155.0(6)<br>145(1)	156.0(9)	147(1)	161.9(4)	143.2(6)	35.3(9)<br>12.6(3)	26.8(6)	161.4(7)	30.2(6)	34.6(7)	166.3(7)
τ_m	39.2(5)	35.5(4)<br>43.9(7)	39.3(6)	37.7(7)	40.5(3)	40.8(4)	24.2(4)<br>18.9(1)	23.2(2)	38.2(5)	23.6(2)	27.6(3)	39.6(5)
Sugar pucker	C2′-*endo*	C2′-*endo*<br>C2′-*endo*	C2′-*endo*	C2′-*endo*	C2′-*endo*	C1′-*exo*	C3′-*endo*<br>C3′-*endo*	C3′-*endo*	C2′-*endo*	C3′-*endo*	C3′-*endo*	C2′-*endo*

Table 3 footnote:

All numerical values in the table are in degrees. The furanose pseudorotation amplitude (τ_m) and phase angle (P) as well as their deviations (values in parentheses) from the ideal (cyclopentane) model were calculated by the method of Jaskolski [2]. The upper/bottom values refer to conformation I/II of the DNA chain in the d(CGCGCG)$_2$–Cr^{3+} structure. Angle definiton: α: O3'(*n*1)-P-O5'-C5' β: P-O5'-C5'-C4', γ: O5'-C5'-C4'-C3', δ: C5'-C4'-C3'-O3', ε: C4'-C3'-O3'-P(*n*+1), ζ: C3'-O3'-P(*n*+1)-O5'(*n*+1), χ: O4'-C1'-N1-C2 (pyrimidines), O4'-C1'-N9-C4 (purines).

Supplementary Table S3. Average base-pair and local base-pair step helical parameters for selected Z-DNA structures in complex with d-block metal cations identified by their PDB codes in parentheses.

	Helical twist Ω_h (°)	Helical rise (Å)	Inclination η (°)	Tip (°)	Tilt (°)	Roll (°)	Shift (Å)	Slide (Å)	Rise (Å)
$d(CGCGCG)_2$–Cr^{3+} (4r15)[a]	-60.94<br>-60.43	3.52<br>3.63	4.33<br>4.03	0.19<br>1.06	-0.40<br>-0.27	-1.56<br>-1.70	-0.02<br>-0.03	3.07<br>3.04	3.52<br>3.51
$d(CGCGCG)_2$–Spk–Mn^{2+} (4hig)	-58.99	4.50	17.45	5.23	0.71	-3.10	-0.07	2.79	3.45
$d(CGCGCG)_2$–Spk–Zn^{2+} (4hif)	-60.93	4.11	15.64	0.47	-0.03	-2.45	-0.14	2.87	3.52
d(CACGCG):d(CGCGTG)–Mn^{2+} (3fq5)[b]	-58.72<br>-60.10	3.99<br>3.94	11.45<br>7.94	-12.58<br>-12.42	-0.76<br>-0.81	-1.46<br>-1.85	-0.02<br>-0.03	2.77<br>2.91	3.62<br>3.69
d(CACGCG):d(CGCGTG)–Mn^{2+} (4dwy)[b]	-57.75<br>-59.34	3.64<br>4.08	3.64<br>18.94	-1.38<br>-8.19	-0.81<br>0.08	-1.52<br>-1.78	-0.04<br>0.03	2.82<br>3.00	3.54<br>3.63
d(CACGCG):d(CGCGTG)–Mn^{2+} (4dy8)	-62.37	2.90	2.38	-14.51	-0.23	-2.01	-0.20	2.89	3.48
$d(CGCGCG)_2$–Cu^{2+} (1d39)	-60.45	4.08	8.48	-1.48	0.04	-2.67	0.02	2.85	3.55

(a) Upper/bottom values refer to two alternate conformations present for the duplex in the asymmetric unit.

(b) Upper/bottom values refer to two duplexes present in the asymmetric unit.

REFERENCES

1 Kabsch W (2010) Acta Cryst D 66:125–132

2 Jaskolski M (1984) Acta Cryst D 40:364–366
